# Prediction of academic achievement based on learning strategies and outcome expectations among medical students

**DOI:** 10.1186/s12909-019-1527-9

**Published:** 2019-04-05

**Authors:** Sakineh Nabizadeh, Sepideh Hajian, Zohre Sheikhan, Fatemeh Rafiei

**Affiliations:** 1 Student Research Committee, Department of Midwifery & Reproductive Health, School of Nursing & Midwifery, Shahid Beheshti University of Medical Sciences, Vali Asr Ave., Ayatollah Rafsanjani Cross Road, Niayesh Complex, Tehran, Zip code: 1985717443 Iran; 2 Department of Midwifery and Reproductive Health, Faculty of Nursing and Midwifery, Shahid Beheshti University of Medical Sciences, Tehran, Iran; 3 Department of biostatistics & epidemiology, Arak University of Medical Sciences, A’lam-Al-Hoda Street, Shahid Shiroodi Street, Arak, Markazi Province Zip Code: 3819693345 Iran

**Keywords:** Academic achievement, Learning strategies, Outcome expectations

## Abstract

**Background:**

One of the most important indicators of the effectiveness of teaching can be the academic achievement of learners, which can be influenced by different factors such as learning methods and individual motivations. The purpose of this study was to determine the ability of predicting academic achievement based on learning motivation strategies and outcome expectations based on a theoretical model.

**Methods:**

This descriptive-analytic study was conducted with the participation of 380 male and female students of nine faculties of medical sciences of Shahid Beheshti University of Tehran. Multi-stage sampling along with the questionnaire of motivational strategies for learning and student outcome expectation scale were used for data collection. The college grade point average (CGPA) of students’ past grades was considered as the academic performance variable. Data analysis was performed using Structural Equation Modeling (SEM) in AMOS software.

**Results:**

The mean score of the structure of learning strategies, motivational strategies, outcome expectations, and students’ GPA did not show significant statistical differences in terms of gender, marital status, residence location, field of study, and educational level. There was a direct and significant relationship between the motivational strategies’ structures (*R* = 0.193, *p* < 0.001) as well as learning strategies (*R* = 0.243, *p* < 0.001) and the CGPA, while there was no relationship between outcome expectations and CGPA. Path analysis revealed that self-regulating learning strategies and motivational strategies can predict the academic achievement of these students.

**Conclusions:**

Considering the importance of active and independent learning among medical students, it is necessary for lecturers to use interactive and student-oriented patterns of teaching. Also, students should become familiar with self-regulating learning skills to better understand the information they receive.

**Electronic supplementary material:**

The online version of this article (10.1186/s12909-019-1527-9) contains supplementary material, which is available to authorized users.

## Background

One of the performance measures of any educational system is the students’ academic achievement. Academic achievement is the ability to prove academic achievement in the acquisition of the planned outcome [1]. Many scholars emphasize the impact of mental and cognitive abilities on academic achievement; however, having high intelligence does not guarantee academic achievement, and individuals need to be aware of their learning styles [2]. The learning styles are methods of learning applied by students in achieving, analyzing, and internalizing their newly acquired knowledge [3].

Students of medical sciences encounter massive information to be learned, and more importantly, to be applied in clinical practice. For this reason, as lifelong learners, they need to utilize efficient learning strategies that work well and make voluminous information durable [4].

The learning strategies differ in effectiveness and practicality, although they have common characteristic including meaningful learning or learning with understanding. This happens when students integrate new acquired information with their existing knowledge. Meanwhile, there are a few learning methods available to medical students to manage all information in mind for academic improvement [5]. With a review of the existing theories on teaching and learning, one can see that individuals behave differently in the same situations. One of the reasons for this situation is their learning strategies, with the extent of employing learning strategies and the underlying individual [2], psycho-emotional, and environmental factors affecting the academic achievement of learners [6].

One of the theories in this field is the Social Cognitive Career Theory (SCCT), developed based on Bandura’s general social cognitive theory to predict the success and performance of individuals with regard to their cognitive, psychological, and behavioral aspects. The SCCT model emphasizes the role of individual abilities, self-efficacy beliefs, outcome expectations, and extrinsic factors in achieving academic or professional success [7]. Accordingly, learning is an active cognitive process in the mind which is influenced by factors such as age, personality traits such as compliance with environmental conditions, attendance in the classroom [8], positive interaction with others [9], intrinsic and extrinsic motivational goals, characteristics of the study approach, and individual self-regulating learning strategies [6].

Self-regulated strategies for learning are defined as the ability to learn based on individual endeavors; cognitive and metacognitive self-regulations are considered as an example of these strategies developed by Bandura [10]. Cognitive learning strategies include mental review, expanding and content-organizing; finally, metacognitive learning strategies include critical thinking, self-learning, organizing, self-controlling, and self-assessment [11].

With regards to these strategies, Zimmerman stated that “learners, rather than relying on lecturers, parents, or other educational authorities, manage their own efforts”, which will lead the learners to adopt their way of studying and improve their performance. Cognitive strategies for learners include mental review, semantic expansion, and information-organizing. On the other hand, individuals may use metacognitive strategies to monitor, guide and, if necessary, modify their cognitive strategies, which include planning, supervising and organizing the learning process [12]. In this regard, self-regulating learners are those who have planning, content-organizing, self-learning, self-controlling, and self-evaluating capabilities [11].

On the other hand, motivational self-regulation refers to the active use of motivational strategies that enhance learning; during the learning process, learners find themselves competent, self-confident, and independent and can plan, organize, self-control, and self-assess for learning [12].

However, the results of research in using cognitive and metacognitive strategies are contradictory regarding the academic achievement of learners, which is attributed to motivational stimuli or individual’s perceptions of their ability or outcome expectations in the future [13, 14]. According to Bandura’s social cognitive theory, the outcome expectations predict behaviors, meaning that these expectations can affect the person’s ultimate behavior using positive motivators or negative consequences that reduce motivation [15].

The results of studies on the role of learning strategies and learning expectations are also contradictory. For example, among students of United Arab Emirates (UAE) universities, self-efficacy and metacognitive strategies were the strongest predictors of academic achievement [16], while in a study in Iran, self-regulating strategies of learning did not show any significant difference [17]. Also, a study on the effect of using self-regulating strategies on the abilities of learners who study through virtual education showed that the use of these strategies alone was not effective in improving their learning [18].

However, studies on the relationship between learning strategies and academic performance of medical students are limited. A review study on self-regulated learning in the medical students’ learning environment suggested that novice students in pre-clinical environment need more support from others, specifically from seniors, to help them formulate learning objectives and handle the new learning environment [19].

The purpose of this study was to determine the predictability of academic achievement based on learning strategies and outcome expectations based on social cognitive theory of Bandura among preclinical students of Shahid Beheshti University of Medical Sciences in Tehran in 2017. With regard to the research goal, the following hypotheses were developed:

1. There is a significant relationship between learning strategies as well as motivational strategies and academic achievement;
2. There is a significant relationship between the outcome expectations and academic achievement;
3. There is a significant relationship between extrinsic individual factors and academic achievement;
4. Learning strategies and outcome expectations can predict academic achievement among medical students.

## Method

### Participants and inclusion/exclusion criteria

This descriptive-analytic study was conducted on a sample of undergraduate female and male students of medical sciences in Shahid Beheshti University of Tehran. The inclusion criteria were as follows:

1- Being an undergraduate student of years 2, 3, or 4 in one of medical sciences fields (pre-clinical for medical students), 2- Based on the self-report of the students, not having any of the following experiences in the last year: separation or divorce (themselves or their parents), death of a family member, addiction to drug and psychotropic drugs, severe illness, or severe family crises. In any stage of the study, in case any participant decided to abandon the collaboration, he/she would have been omitted from the study.

### Sampling and sample size

Sampling was done as multi-stage. Initially, the total population of undergraduate students in nine faculties of Medical Sciences of Shahid Beheshti University of Tehran was obtained from the Academic Administration Deputy (*N* = 2857). Then, each faculty was considered as a cluster and based on each cluster’s population, a quota was allocated to them. In this step, a quota sampling procedure with field of study was applied as the main quota of the control variable. Finally, convenience sampling was performed on each faculty from eligible students until the specified sample size was over. The initial sample size was calculated using the Cochran formula as below:

$$ {\displaystyle \begin{array}{l}\mathrm{N}=\frac{\frac{{Z_{\alpha}}^2 pq}{d^2}}{1+\frac{1}{N}\left(\frac{{z_{\alpha}}^2 pq}{d^2}-1\right)}=\frac{\frac{\Big({1.96}^{2\Big)}0.5\times 0.5}{(0.05)^2}}{1+\frac{1}{2857}\left(\frac{\Big({1.96}^{2\Big)}0.5\times 0.5}{(0.05)^2}-1\right)}=339\\ {}\alpha =0.05.\mathrm{d}=0.05,\mathrm{p}=0.5,\mathrm{q}=0.5,\mathrm{Z}\upalpha =1.96,\mathrm{N}=2857\end{array}} $$

The final sample size was calculated to be 380 people considering a loss of 10% and a significance level of less than 0.05.

### Study instruments

Since the theoretical framework of this research was based on the SCCT performance model, the most important structures of this model were extracted including cognitive learning abilities, self-efficacy beliefs, outcome expectations, as well as individual goals and factors. Then, their relationship with students’ academic achievement was investigated through appropriate tools. A self-administered questionnaire, consisting of demographic information, learning strategies, and outcome expectations, was used based on the study objectives.

The first section of the questionnaire evaluated the extrinsic individual factors, i.e. demographic characteristics, and variables indicating intrinsic goals such as previous interest and satisfaction with the field of study, study hours per week, and hours spent for work other than study.

The second section, i.e. motivational strategies for learning questionnaire (MSLQ), was used in its 81-item form in this study. This scale was first developed by Pintrich and his colleagues in 1990 for assessing college students’ motivational orientations and their utilization of different learning strategies for teachers or researchers in education [20]. In addition, The MSLQ may be adapted to the researcher’s or teacher’s need on how to enhance their students’ levels of motivation and learning strategies. Also, academic members can use the MSLQ to receive feedback on their students and to support them regarding course adjustments. Meanwhile, students can also use it for self-assessing their abilities and weaknesses in their courses [21].

The MSLQ is based on the general cognitive and social cognitive theory of Bandura regarding motivational and learning strategies. This instrument has essentially two sections: i) motivation section; ii) learning strategies. The first section consists of 31 items which assess:

1. students’ goals and value beliefs for a course, as *value component*, with 14 items including intrinsic orientation (4items), extrinsic evaluation (4 items), and task value (6 items);
2. students’ beliefs about their skill to succeed along with their anxiety about tests in a course, as *expectancy component,* with 17 items including, controlling learning beliefs (4 items), self-efficacy (8 items) and test anxiety (5 items).

The second section includes 50 items on

1. students’ use of different self-regulated strategies, as *cognitive and metacognitive strategies,* with 31 items including rehearsal (4items), elaboration (6items), organization (4 items), critical thinking (5 items) and metacognitive self-regulation (12 items);
2. student management of different resources, as *resource management strategies,* with 19 items including time and study environment (8 items), effort regulation (4 items), peer learning (3 items), and seeking help (4 items).

Students rate themselves on a seven-point Likert scale from “not at all true of me” to “very true of me.” Scales are constructed by taking the mean of the items making up that scale. Scores can range from 81 to 567 in total. However, Pintrich and his colleagues did not provide norms for the MSLQ; rather it has been designed to be used at the course level. They assumed that students’ responses to the questions could vary and the same individual might report different levels of motivation or strategy use depending on the course [20].

In the third section, student outcome expectations scale (SOES) was used. It was designed and developed by Betz and Voyten in 1997 to determine students’ beliefs concerning the performance of a behavior. The original version of SOES consists of 13 items defined in three factors. This scale measures students’ responses in a 4-point range on a Likert scale [22].

The psychometric properties analysis of the MSLQ has previously been performed, and the overall internal consistency reliability, provided by Pintrich et al., has been found to be sufficient (more than 0.7 for two main sections) [21]. In addition to English, the MSLQ has been translated into other languages [23] including Persian [24]. The reliability of this instrument has been confirmed, and its internal consistency have proved to be acceptable for all MSLQ items together (Cronbach’s alpha ≥0.80) and every individual domain (Cronbach’s alpha ≥0.70) [25].

The psychometric properties analysis of the SOES in an Iranian methodological study has been confirmed by confirmatory factor analysis through the Principal Component Analysis of 11 items and 4 factors (Table 2). The Cronbach’s alpha coefficients across the four sub-scales were between 0.65 and 0.79, indicating that the questionnaire had acceptable internal consistency [26].

### Data collection

After explaining the goals of the study and obtaining written informed consent letter from the students, the questionnaires were provided to them. At the beginning of administration of the questionnaires, the researcher (first author) explained about each section of the instrument in detail and emphasized the importance of students’ responses that can offer helpful suggestions to the students on how to enhance their levels of learning motivation and strategies. Further, if necessary, they could receive feedback from the researcher. In addition, they were informed that they would not be marked or judged at all; rather the result could be adapted to the instructor’s academic educational needs. So, they were asked to rate themselves by answering the whole items in papers completely and not to leave any item unanswered. In case only less than 5% of the items were not answered, the score of “not sure” option would have been assigned to those items, while if more than 5% of the items were left unanswered, that questionnaire would have been omitted from the study.

The questionnaires were administered in regular classrooms. The completion of the questionnaire took an average of 20 min per person. The CGPA in all past semesters was considered as the variable of academic achievement. Data collection was performed within 2 months.

### Statistical analysis

To test the research hypotheses, raw data were analyzed using SPSS 22 and then entered into the AMOS software. Using structural equation modeling (SEM), the relationship between the studied variables and the students’ academic achievement was investigated. The significance level was considered to be less than 0.05 (Additional file 1).

## Results

### Descriptive information

In this research, 380 students from different fields of medical sciences were studied. The response rate was 100% and no questionnaires were dropped. The average age of respondents was 21.35 ± 2.91 years, with the youngest being 18 and the eldest being 47 years old. Some demographic features of the participants are presented in Table 1. Comparison of the CGPA of students did not show any significant difference in terms of the variables of gender, field of study, educational level, marital status, employment status, and place of residence.

**Table 1:** Demographic characteristics of the participants (*N* = 380)

characteristics	n	Percent (%)
gender		
female	229	60.3
male	151	39.7
grade		
Second	168	44.2
Third	158	41.6
Forth and more	54	14.2
Field of study		
Medicine	87	22.9
Dentistry	51	13.4
Pharmacy	32	8.4
Paramedicine	143	37.6
Midwifery	17	4.5
Nursing	17	4.5
Anesthesiology	16	4.2
Operation room	17	4.5
Marital status		
Single	359	94.5
Married	20	5.2
other	1	0.3
residence		
parents’/own home	170	44.8
dormitory	208	54.7
Relatives’ home	2	0.5
Second vocation		
Yes	49	12.9
no	331	87.1

The distributions of the average scores for each subscale of the questionnaires and the CGPA of students in the previous semesters are reported in Table 2.

**Table 2:** Distribution of questionnaires’ scores and their correlation coefficients to students’ grade point average (GPA)

Variable	Mean ± (S,D)	Min & Max	Default Min & Max	R-correlation coefficient
MOTIVATIONAL STRATEGIES	157.62 (41.35)	31–217	31–217	0.193^b^
Value component	71.9 (15.76)	19–98	14–98	0.201^b^
Intrinsic orientation	21.08(5.19)	4–28	4–28	0.162^b^
Extrinsic evaluation	19.18 (5.41)	4–28	4–28	0.109^a^
Task value	31.12 (7.13)	9–42	6–42	0.156^b^
expectancy component	85.79 (13.24)	17–119	17–129	0.150^a^
Control of Learning Beliefs	22.57 (4.05)	4–28	4–28	0.033(N.S)
Self-Efficacy	41.92 (8.36)	8–56	8–56	0.294^b^
Test Anxiety	21.11 (6.05)	5–35	5–35	- 0.112^b^
LEARNING STRATEGIES	221.62 (38.35)	101–340	5–350	0.243^b^
Cognitive and Metacognitive Strategies	138.45 (28.36)	46–211	31–217	0.244^b^
Rehearsal	16.62 (4.98)	4–28	4–28	0.193^b^
Elaboration	27.62 (6.9)	6–42	6–42	0.228^b^
Organization	18.46 (5.4)	4–28	4–28	0.230^b^
Critical Thinking	22.78 (5.73)	5–35	5–35	0.207^b^
Metacognitive Self-Regulation	52.95 (10.05)	27–78	12–84	0.196^b^
Resource Management Strategies	83.17 (16.33)	19–129	19–133	0.190^b^
Time and Study Environment	36.79 (6.56)	8–52	8–56	0.124^a^
Effort Regulation	16.51 (4.04)	4–28	4–28	0.043(N.S)
Peer Learning	11.57 (4.15)	3–21	3–21	0.177^b^
Help Seeking	17.72 **(**4.35)	4–28	4–28	0.186^b^
OUTCOME EXPECTANCY	33.76 (6.71)	11–43	11–44	0.049(N.S)
Future orientation	12.39 (3.00)	4–19	4–16	0.048(N.S)
Job satisfaction	9.39 (2.62)	3–12	3–12	0.057(N.S)
Personal expectations	5.97 (1.94)	2–8	2–8	−0.048(N.S)
Personal trust	7.00 (1.50)	2–8	2–8	0.084(N.S)
Vacation hours/week	20.88 (2.47)	–	–	- 0.360^a^
Study hours/week	59.90 (3.50)	–	–	0.256^b^
Grade point average (GPA)	16.42 (1.30)	12–19.15	0–20	–

^a^less than 0.05, ^b^ less than 0.001

A review of the distribution of these variables indicated normal data distribution. The average scores of the structures of learning strategies, motivational strategies, outcome expectations, and their sub-structures as well as the CGPA of students did not show significant statistical differences in terms of gender, marital status, permanent and current place of residence, field of study, and educational level.

### Testing the Study’s hypotheses

The calculation of Pearson correlation coefficients between study variables and the CGPA of students (*P* < 0.05) revealed a direct linear and positive correlation between the motivational strategies and learning strategies plus most of their sub-structures and CGPA students, confirming the first hypothesis of the study. However, there was no such relationship between the outcome expectations and the CGPA. Therefore, the second hypothesis of the research is rejected.

Also, regarding the third hypothesis of the study, merely a significant relationship was confirmed between hours of study per week (positive linear) and hours spent on work other than education (negative linear) with CGPA. However, the CGPA of students showed no significant difference in terms of age, gender, parent’s education level, marital status, employment status (other than education), field of study, previous interest in the field of study, and satisfaction with the field of study (Table 2).

In order to test the fourth hypothesis and to determine the accuracy of the conceptual model of the research, considering the available data, structural equation modeling using the maximum probability method was utilized with the help of AMOS software.

When examining the structural equation, the fitness between the hypothesis model and study data is an important component. Accordingly, the goodness of fit indices was evaluated. The results in Table 3 indicate that this model is acceptable in terms of all indicators of goodness of fit and research data.

**Table 3:** Indices of goodness of fit for conceptual model of the research

Fitness criteria	approximate fit indices	Model’s index value
CMIN^a^	0 < CMIN<2df	82.311
df^b^		37
P^c^	0.01 < p ≤ 0.05	0.001
CMIN/df	0 < CMIN/df < 2	2.225
RMSEA^d^	0 ≤ RMSEA ≤0.05	0.057
P (RMSEA< 0.05)	0.1 < *p* ≤ 1	0.232
GFI ^e^	0.95 ≤ GFI ≤ 1	0.961
AGFI^f^	0.9 ≤ AGFI≤1	0.930
NFI^g^	0.95 ≤ NFI ≤ 1	0.964
TLI^h^	0.97 ≤ TLI ≤ 1	0.969
IFI^i^	> 0.9	0.980
CFI^j^	0.97 ≤ CFI < 1	0.979
RFI^k^	> 0.6	0.946

^a^Q-Squared, ^b^degree of Freedom, ^c^Level of significance, ^d^Root Mean Square Error of Approximation, ^e^goodness of fit index, ^f^ adjusted goodness of fit index ^g^Nonnormed Fit Index, ^h^ Tucker-Lewis Index, ^i^ Incremental Fit Index, ^j^comparative fit index, ^k^relative fit Index

**Fig. 1:**
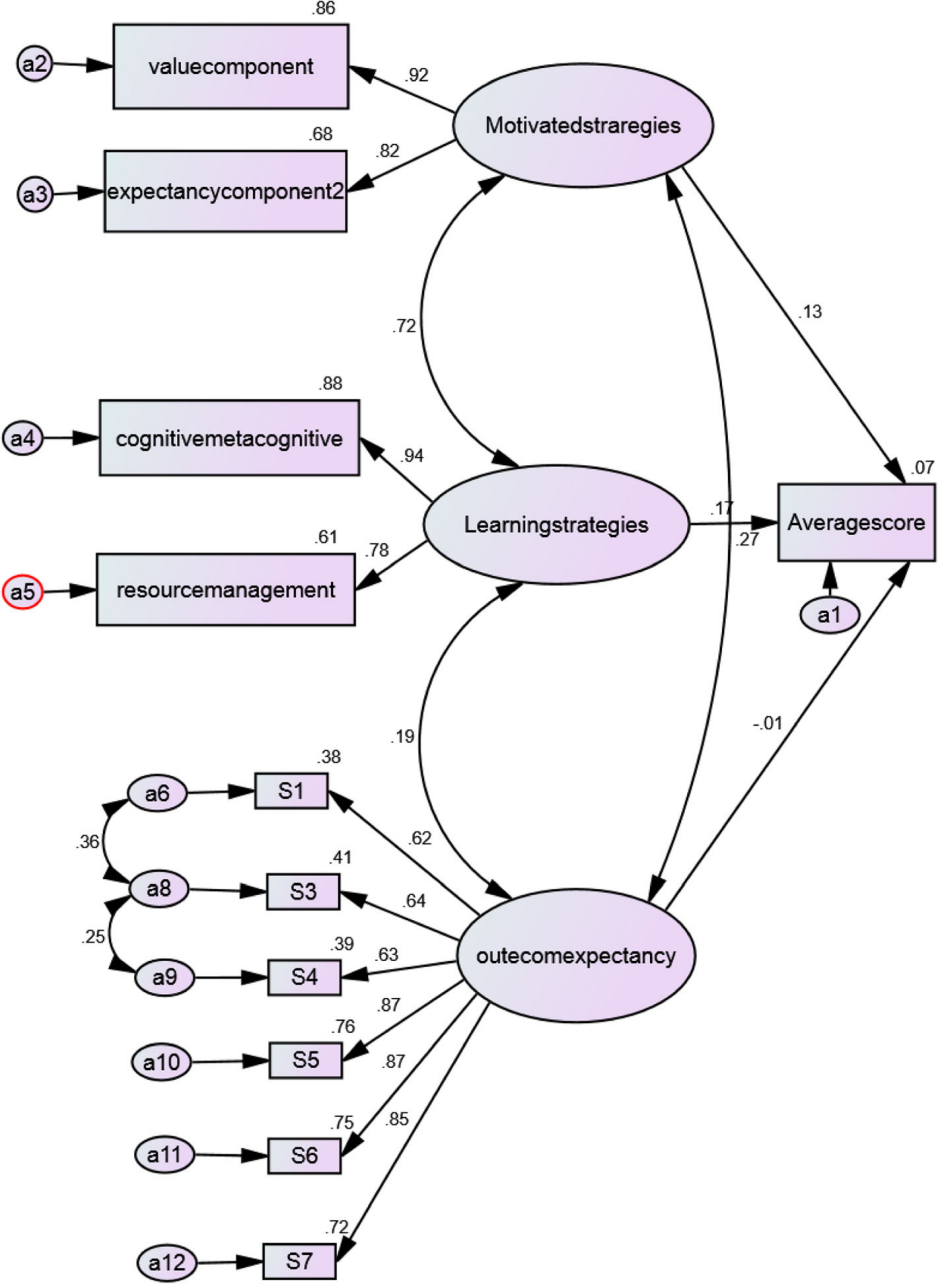
structured relationships model between motivated strategies, learning strategies, outcome expectations and students’ college grade point average (GPA)

In Fig. 1, the conceptual model of the research, along with standard factor loads, shows the relationship between learning strategies, motivational strategies, as well as outcome expectations and the CGPA of students, indicating that among these three variables, the learning strategies directly affect the CGPA.

In this regard, the direct effects of research variables and their significance for each of the direct paths of the conceptual model of the research were examined. The results revealed that among the three main variables, i.e. learning strategies, motivational strategies, and outcome expectations, the greatest impact belonged to learning strategies. In other words, having examined the simultaneous effect of each of these variables as an independent variable on the CGPA, the standard coefficients of the model indicated that the variable of learning strategies has a greater effect on the increase in the CGPA and, in turn, on academic achievement of the students (Table 4).

**Table 4:** significance analysis of path coefficients of directed effect on study conceptual model

path			Non-standardized factor loading	standardized factor loading	Standard error estimation	T -value	*p*-value
Motivated strategies	➔	Expectancy component	1	0.823			
Motivated strategie	➔	Value component	1.504	0.925	0.08	17.093	0.0001
Learning strategies	➔	Resource management	1	0.784			
Learning strategies	➔	Cognitive metacognitive	2.364	0.937	0.154	15.339	0.0001
Outcome expectancy	➔	S1	1	0.619			
Outcome expectancy	➔	S3	1.001	0.640	0.076	13.206	0.0001
Outcome expectancy	➔	S4	1.046	0.626	0.101	10.374	0.0001
Outcome expectancy	➔	S5	1.416	0.872	0.107	13.181	0.0001
Outcome expectancy	➔	S6	1.366	0.866	0.104	13.132	0.0001
Outcome expectancy	➔	S7	1.382	0.846	0.107	12.947	0.0001
Learning strategies	➔	Average score	0.019	0.167	0.010	1.965	0.049
Outcome expectancy	➔	Average score	1.028	0.013	0.120	−0.232	0.817
Motivated strategies	➔	Average score	0.019	0.129	0.013	1.481	0.139

In addition, the study of direct and indirect effects of the model demonstrated that the greatest effect belonged to cognitive and metacognitive strategies, followed by the variable of valuation, which has an indirect positive effect on academic achievement (Table 5).

**Table 5:** The sum of the direct and indirect effects of the study’s model on students’ academic chievement

path			Direct effects	Indirect effects	Total effects
Outcome expectancy	➔	Average score	- 0.013	0	- 0.013
Learning strategies	➔	Average score	0.167	0	0.167
Motivated strategies	➔	Average score	0.129	0	0.129
value component	➔	Average score	0	1.054	1.054
expectancycomponent2	➔	Average score	0	0.952	0.952
Resource management	➔	Average score	0	0.951	0.951
cognitive metacognitive	➔	Average score	0	1.104	1.104
Value component	➔	Motivated strategies	0.925	0	0.925
expectancycomponent2	➔	Motivated strategies	0.823	0	0.823
Resource management	➔	Learning strategies	0.784	0	0.784
Cognitive metacognitive	➔	Learning strategies	0.937	0	0.937

## Discussion

The findings of this study suggested that cognitive and metacognitive learning strategies and motivational strategies are predictors of academic achievement of students. In other words, students who use self-regulating and motivational learning strategies have a higher academic performance. Stating that there is a significant relationship between academic achievement and use of self-regulating learning strategies, Zimmerman suggested that although most learners use these strategies for learning, what distinguish them from each other are their awareness of how to use them and having a motivation for using them [12]. A review of 14 articles assessing self-regulated strategies of learning indicated that this type of learning is associated with academic achievement and success in clinical skills in the future [19].

Students participating in this study had above average levels of learning strategies and motivational strategies. It suggests that students try to actively learn information, and experience and direct their own learning, instead of relying on the classroom and instructional environment. One of the reasons can be the nature of educational curriculum in medical sciences and the critical role of clinical professions in dealing with the health of patients. In addition to focusing, practicing and planning, self-learning, memorizing, and mentally reviewing tips and points and asking for peer help in obtaining favorable grades in exams, it is necessary for these students to apply this information in real-life context and to obtain clinical competencies. These findings are consistent with Mukhtar et al.’s results who concluded that the assistants in different medical fields who feel more attached to their profession and, as a result, to other people, use more self-regulation skills for learning and had a higher academic performance [27].

Among the SCCT structures examined, the students’ academic achievement in this study was most influenced, in terms of self-regulating learning strategies, by the changes in organizing and expansion strategies, which is in line with the results obtained by Muwonge et al. (2018). They stated that the organizing strategy is one of the best and most complete types of learning strategies [14]. In addition, the strategy of semantic expanding through addition of new information to link to previous information should always be considered by lecturers in teaching skills.

Furthermore, motivational strategies indirectly played an effective role in the student’s academic achievement in this study. This finding suggests that students, in addition to having an interest in academic education, had a great incentive to enter their field of study; however, only motivation cannot predict the proper academic performance. In line with the findings of this study, Muwonge et al., in their study on the self-regulation and motivational learning strategies among 1081 students from seven universities in Uganda, stated that motivational strategies influenced students’ academic achievement only through affecting critical thinking strategies and organizing skills. Therefore, educational interventions to improve the academic performance of students should focus on increasing the motivation of learners and enhance their use of cognitive learning strategies [14]. Thus, it can be said that students who have a high motivation to obtain a better score demonstrate more effort, better organize their information, have better time management, and show better performance [27].

In a study by Wibrowski, the freshmen students undergoing Skills Learning Support Program (SLSP) had a higher level of study and motivation skills and better academic achievement indicators compared to their peer students who were not involved in this program [1]. Griffin et al. suggested that awareness of metacognitive strategies and promotion of study skills have a positive effect on academic achievement and intrinsic motivation as self-regulating learners will be able to use different means to acquire active learning experiences and, whenever necessary, organize learning strategies according to their requirements, task features, and context-specific conditions [28].

The findings of this study suggested that the effect of self-efficacy beliefs on academic achievement is significant and worthy of discussion. The findings from two different studies on medical students in Turkey suggested that most medical students used self-regulation skills and believed in their ability to learn effectively [29, 30]. [23, Further, Kek and Huijser concluded that higher levels of self-efficacy are associated with utilization of higher levels of learning strategies [31]. On the other hand, in a study by Nei et al., the self-efficacy was a negative predictor of stress of exam, which indicated that students who perceived an educational situation as stressful were less likely to be able to rely on their own abilities to control that situation [32].

In accordance with the SCCT performance model, self-efficacy beliefs first play an important role in the growth of intrinsic motivation, and contributing to facilitating the cognitive processes. Indeed, their improvement increases the probability of using cognitive strategies and, thereby enhancing the level of student performance. In a study by Navaro, self-efficacy was an important component in predicting the academic satisfaction and achievement of engineering students regardless of their demographic characteristics [33]. The results of meta-analysis of 167 studies on determining the factors affecting the academic achievement of students showed that self-efficacy, among other structures of learning strategies, had the strongest relationship with the CGPA of students [34].

According to SCCT, self-efficacy had a significant effect on outcome expectations. In other words, the outcomes that people expect are related to their judgment of their ability to perform their tasks. Individuals with high self-efficacy tend to be more inclined to visualize positive outcomes about their tasks. The results of a study by Lent on evaluating the factors influencing the interest in education among 600 Portuguese students suggested that self-efficacy and outcome expectations together predict interest in education, which mediates the relationships between self-efficacy and outcome expectations in choosing the future profession [7]. However, in the present study, outcome expectations were not a significant component in predicting students’ academic achievement. The reason for this can be partially related to the negative expectations of students from the outcome of their field of study and the reduction of job opportunities due to the mismatch between student admissions and the needs of the community.

In this study, different learning strategies and outcome expectations did not show significant differences in terms of personal characteristics such as gender and environment, consistent with Turan et Al. and Demirören et al. studies in Turkey [29, 30].

Gudaganavar et al. in a study on the study habits of 250 Indian students found that although there was no significant difference in the general habits of study between male and female students, women were significantly different from men in aspects such as taking notes at the time of study, organizing information, and preparing for tests. These habits had a direct relationship with women’s academic achievement, while the use of such methods in men did not show any significant relationship with their academic performance [35].

Farooq et al. studied the learning approaches and CGPA of students. They found that females were better than males and their academic achievement had a direct relationship with the educational level of their parents [36]. Considering contradictory results of several studies in this area, further studies might give better information about the effect of personal differences including gender differences such as the role of cultural norms.

The present study’s findings indicated that the increase in study hours per week and employment of students in jobs had a positive and negative impact on their academic achievement, respectively. This suggests that the quality of study and the focus on the course can directly improve the academic performance, with the simultaneous employment having a negative impact on student performance through reducing the hours of study. On the other hand, in spite of the effective role of extrinsic factors in students’ academic performance, none of the personal variables studied in this research were related to the student’s academic achievement. This indicates that learning strategies and motivational strategies have a role to play in determining the academic achievement of students regardless of their individual and social characteristics.

The present study for the first time investigated the academic achievement of medical students based on the SCCT theoretical model. However, as with any other research, there were some limitations. Firstly, the collected data were based on a cross-sectional design and a non-probable sampling. Secondly, as the data were gathered from a sample of one state university in Iran, thus, generalizability of the findings of this study may be limited to the target population. Finally, in spite of complete response rate to our study questionnaires, we did not explore such academic achievement’s determinants of students who did not or refused to participate in this study, therefore, this problem should be considered in future studies. These limitations suggest that the study findings ought to be interpreted cautiously.

## Conclusions

Knowing the cognitive and metacognitive strategies in student learning skills and identifying the most important motivational factors and goals in the success of their academic achievement can contribute to better understanding of these strategies and factors affecting the performance of students by lecturers and educational planners. Further, all faculty members and lecturers in the medical education should be familiarized with the ways to enhance active learning skills and be encouraged to involve students in the teaching-learning process and to use interactive teaching patterns. Therefore, medical teachers should recognize their students’ motivations and prevailing learning strategies, monitor their learning in their academic environment, and encourage them to be engaged in learning.

## Additional file

Additional file 1: Raw data. (SAV 78 kb)

## Abbreviations

CGPA: College grade point average
MSLQ: Motivational strategies for learning questionnaire
SCCT: Social Cognitive Career Theory
SEM: Structural Equation Modeling
SLSP: Skills Learning Support Program
SOES: Student outcome expectations scale
UAE: United Arab Emirates
